# Phenotypic Parent Selection Within a Khorasan Wheat Collection and Genetic Variation in Advanced Breeding Lines Derived by Hybridization With Durum Wheat

**DOI:** 10.3389/fpls.2019.01460

**Published:** 2019-11-20

**Authors:** Anna Iannucci, Pasquale Codianni

**Affiliations:** Centro di ricerca Cerealicoltura e Colture Industriali, Consiglio per la ricerca in agricoltura e l’analisi dell’economia agraria (CREA-CI), Foggia, Italy

**Keywords:** agronomic traits, Khorasan germplasm, durum wheat, genetic diversity, genetic resources, interspecies crosses, phenotypic diversity

## Abstract

Tetraploid relatives of durum wheat (*Triticum turgidum* L. subsp. *durum* (Desf.) Husnot) represent an important reservoir of economically useful genes for development of new wheat cultivars. Two field experiments were conducted at Foggia (Italy), in the 2004 to 2006 and 2012 to 2015 growing seasons. In the first, 77 Khorasan wheat [*T. turgidum* subsp. *turanicum* (Jakubz.) Á. Löve & D. Löve] accessions from 23 countries of four geographic regions (Africa, Asia, Europe, and others) were evaluated to explore breeding opportunities. Seven agronomic traits were used to describe the diversity among the accessions: days to heading (HT), plant height (PH), grain yield (GY), specific weight (SW) as an indication of the density of the grain, 1000-grain weight (TGW), protein content, and gluten content. The total Shannon–Weaver diversity index was used to estimate phenotypic diversity, which ranged from monomorphic for PC (0.39) to highly polymorphic for TGW (0.67). A high level of total variation (87%) was attributed to the within-region diversity. The accessions grouped into six clusters, and seven elite accessions were selected as parents for crosses with durum wheat. In the second experiment, ten parents (seven Khorasan accessions and three durum wheat cultivars) and 790 F6 recombinant inbred lines (RILs) from the different *T. durum* × *T. turgidum* subsp. *turanicum* crosses were included to study genotypic and phenotypic variability of the same agronomic traits, plus the susceptibility index (SI) for disease. The genotypic coefficients of variation were lower than the phenotypic ones for all of the traits, which showed an environment effect on expression of these traits. High broad-sense heritability (h^2^_b_ > 86%) was recorded for all traits, and high h^2^_b_ coupled with high genetic gain as percentage of the mean (ΔG) was observed for HT, PH, GY, and SW, and for SI. This suggests that selection for these traits will provide good responses. Four principal components explained 70% of the total variation, and the genotypes were clustered into 20 groups. According to the results, some lines could be tested in varietal registration trials, and/or could be used as a significant breeding pool for durum wheat cultivar development in Mediterranean area.

## Introduction

Durum wheat (*Triticum turgidum* subsp. *durum* (Desf.) Husnot, 2n = 4x = 28, AABB) is a staple crop that is adapted to the hot, dry conditions of the Mediterranean Basin and to similar climates in other parts of the world, with Mediterranean countries accounting for ∼75% of the world durum wheat production (Shewry and Hey, 2015). In Italy, durum wheat production is concentrated in the southern regions (∼67%), and it is mainly used for pasta (D’Egidio, 2007).

As stated by the FAO (2016), by 2050, the world demand for wheat will be 40% greater due to population growth, climate change, rising incomes in emerging countries, and increasing urbanization. In particular, to meet the increasing demand for durum wheat in the future in the Mediterranean region, grain production must increase at a rate of about 5.85% from 2017 to 2023 (https://www.marketresearchfuture.com/reports/pasta-market-2428). For these reasons, new wheat varieties that can adapt to future challenges must be developed. More efficient use of biodiversity in breeding programs is essential to this progress (Mackay et al., 2016).

Improvement depends on a continuous supply of new germplasm as donors of various genes of agronomic importance. Future gains in yield potential and quality standard of products are desirable and can be achieved by exploiting the genetic diversity of related cereal species (Mengistu et al., 2016b) that are predicted to contain the highest numbers of unexploited, but potentially useful, alleles (Laidò et al., 2013). In this context, landraces, wild forms, and other related wild species are potential sources of useful alleles for durum wheat improvement. These will facilitate selection for high levels of recombination, and thus allow the generation of new and more adapted genotypes (Lopes et al., 2015). The use of related cereal species that have the same genome as the current wheat germplasm for direct crossing and introgression of adaptive traits represents an attractive breeding strategy.

The tetraploid relatives of durum wheat include *T. turgidum* subsp. *turanicum* (Jakubz.) Á. Löve & D. Löve (2n = 4x = 28, AABB), which is commonly known as Oriental or Khorasan wheat. It is a neglected and underutilized species, which appears to have survived over the centuries in subsistence farming systems in the Near East, Central Asia, and Mediterranean areas (Grausgruber et al., 2005; Rodríguez-Quijano et al., 2010). It has a stem with thin straw, large spikes, and very long kernels, which are glassy with an intense amber color. In particular, the grain are large, and the 1,000-grain weight can reach 50 g to 60 g. Khorasan also has high protein content and important nutritional qualities (Sissons and Hare, 2002; Laddomada et al., 2017). In terms of the ease of gene transfer to durum wheat, Khorasan wheat represents a desirable donor for potential improvement. The genetic diversity estimated by simple sequence repeats and diversity array technology markers on 20 accessions of Khorasan wheat suggested the genetic potential for detection of unexplored alleles, with the possibility to use some genotypes as parents to develop high-yielding durum wheat lines in breeding programs (Laidò et al., 2013). Similar results were found by Khlestkina et al. (2006).

However, there is little or no information available on partitioning of the variability and estimation of the genetic gains of the agronomic traits of Khorasan wheat. As this species is grown throughout countries with a wide range of variations in altitude, rainfall, temperature, agricultural systems, and socio-economic factors, for efficient use of the collected germplasm in cereal breeding programs it is essential to determine the trait variability and distribution among and within the different geographic regions.

To detect the presence of variants of potential interest for breeding purposes, the evaluation of the diversity in the gene pool and the characterization of available accessions of Khorasan are necessary. A number of methods are available for analysis of genetic diversity in germplasm accessions, breeding lines and populations. The regional or geographic phenotypic diversity within a germplasm collection can be determined by the Shannon-Weaver (*H′*) index (Grenier et al., 2000) based on the frequency data. Furthermore, multivariate analysis methods, including principal component analysis (PCA) and cluster methods are also useful tool to identify groups with desirable traits for breeding (Bertan et al., 2007). These statistical methods have been used previously to determine the extent of genetic diversity for various morphological, agronomic, and physiological traits in durum wheat under different environments (Ruiz et al., 2012; Lopes et al., 2015; Alsaleh et al., 2016). According to Mansouri et al. (2018), the diversity of the advanced breeding lines derived from the segregating generations of interspecific crosses need to be evaluated to determine the genetic gain that can be expected through the selection of any given trait and to investigate the potential production of superior recombinant genotypes.

In this context, a breeding plan was developed to initially analyze the degree of similarity/dissimilarity in a Khorasan wheat collection from different eco-geographic regions of the world. The aim of the experiments was to (i) select superior genotypes of Khorasan wheat to be used as parents to be crossed with three durum wheat lines and (ii) introgress these genes to enlarge the genetic diversity and to select superior genotypes.

## Material and Methods

### Plant Materials

#### Collection Population

Seventy-seven accessions of Khorasan wheat that were collected from different eco-geographic regions of the world and maintained by the Research Centre for Cereal and Industrial Crops (CREA-CI; Foggia, Italy). Seed samples were kindly provided by the Institute of Biosciences and Bioresources gene bank (Bari, Italy).

The genotypes used in this study were developed through pure-line selection from the available germplasm and sown for three consecutive years, 2005 to 2007. Based on their 23 countries of provenance, the 77 accessions were assigned to 10 groups according to their geography: East Africa (1), North Africa (8), North America (1), Central Asia (9), western Asia (40), Australia (1), eastern Europe (6), southern Europe (6), western Europe (1), and unknown origin (4) (**Table 1**).

**Table 1:** The 77 germplasm accessions of Khorasan wheat used for the evaluation (collection population).

Continent	Subcontinent	Country of origin	No. of accessions	Accession code^†^
Europe	Western	UK	1	PI330552
	Southern	Italy	1	PI278350
		France	1	PI306665
		Portugal	2	PI184526, PI184543
		Spain	2	PI190973, PI256034
	Eastern	Hungary	3	PI272601, PI272602, PI290530
		Poland	1	PI286069
		Romania	1	PI362067
		Ukraine	1	PI251925
Africa	Northern	Egypt	1	PI10391
		Morocco	7	PI185192, PI185193, PI191599, PI192641, PI192658, PI525355, PI559976
	Eastern	Ethiopia	1	CItr14599
Asia	Western	Azerbaijan	3	PI68287, PI68293, PI352514
		Iran	26	PI113392, PI254204, PI254206, PI254209, PI254210, PI254212, PI352515, PI623629, PI623641, PI623656, PI624207, PI624208, PI624209,PI624217, PI624420, PI624421, PI624422, PI624429, PI624892, PI624893, PI625164, PI625187, PI625189, PI625214, PI625401, PI627211
		Iraq	1	PI113393
		Syria	1	PI182717
		Turkey	9	PI115815, PI166308, PI166450, PI166554, PI166959, PI167481, PI254213, PI537992, PI576854
	Central	Afghanistan	7	PI127106, PI317491, PI317495, PI321737, PI321743, PI337643, PI347132
		Turkmenistan	1	PI68104
		Russia	1	PI115814
Australia	Australia	Australia	1	PI67343
North America	USA	USA	1	CItr11390
Unknown	Unknown	Unknown	4	CItr14082, CItr14095, PI210386, PI225331
Total			77	

^†^Gene Bank identification number.

#### RIL Population

Seeds from F1 hybrids derived from 21 crosses of three durum wheat cultivars and seven Khorasan genotypes were used as the starting materials to develop RILs that segregated for high grain yield, specific weight, 1000-grain weight, and superior quality traits. The three durum wheat cultivars were “Iride” (an early heading, short plant height and medium high specific weight cultivar), “Saragolla” (an early heading, medium plant height and high, excellent gluten quality cultivar), and “PR22D89” (a medium early heading, medium tall plant height and high specific weight cultivar). These were released in 1996, 2004, and 2005, respectively. The seven Khorasan genotypes were PI67343, PI525355, PI330552, PI10391, PI68287, CItr14599, and PI115815. Durum wheat and Khorasan genotypes were used as female and male parents, respectively. The F1-crosses were single backcrossed to durum wheat and Korasan genotypes used as the recurrent male parents (**Supplementary Table S1**).

A population of 790 lines was maintained at the CREA-CI (Foggia, Italy) by selfing, starting from the F_2_ generation. All individual F_2_ plants were advanced to the F_6_ generation by the single seed descent method. The F_6_ recombinant inbred lines (here called genotypes) were used for the study of the genetic parameters for the qualitative and quantitative traits.

### Field Trials

Two field experiments were conducted at the CREA-CI (Foggia, southern Italy; 41°28′N, 15°34′E; 76 m a.s.l.), the collection experiment and the breeding population experiment. In the collection experiment, the 77 Khorasan wheat were evaluated over three growing seasons (2004 to 2006), and in the breeding population, 790 RILs and 10 parents were evaluated over three successive growing seasons (2013 to 2015).

Based on 15-year average meteorological data (2000–2014), the climate through the plant growth season (November–June) was characterized by mean precipitation of 357 ± 12 mm yr^-1^, mean minimum and maximum temperatures of 7.6 ± 4.0°C and 18.3 ± 6.3°C, respectively, and annual mean rainfall and temperature of 506 mm and 15.8°C, respectively. These data were obtained from an on-site weather station.

The trials were performed in a clay–loam soil (Typic Chromoxerert) with clay, 36.9%; silt, 50.5%; sand, 12.5%; pH 8 (in H_2_O), available phosphorous (Olsen method), 15 mg kg^-1^; exchangeable potassium K(NH_4_Ac), 800 mg kg^-1^; total nitrogen, 1.4 g kg^-1^; and total organic matter (Walkey–Black method), 21 g kg^-1^. The experiments were arranged in a randomized complete block design, with three replications. The plot area was 5.06 m^2^, which contained 8 rows that were 3.7 m long and 0.17 m apart. The experimental blocks were separated by 1.5 m walkways.

For both experiments, standard cultural management practices were applied. The previous crop was fallow, which was ploughed, hoed, and harrowed twice prior to the wheat planting. Sowing was performed with a plot driller at a seeding density of 350 viable seeds m^-2^. For the collection, this was carried out on December 21, 2003, December 14, 2004, and December 1, 2005. For the breeding population, this was carried out on November 29, 2012, December 7, 2013, and November 20, 2014.

The fertilizer used at sowing was 200 kg ha^-1^ 18/46 fertilizer (elemental N, 18%; P_2_O_5_, 46%; by weight), and at plant tillering 200 kg ha^-1^ NH_4_NO_3_ (elemental N, 26%). Weed control was carried out at the end of tillering, using Buctril Universal (2,4-dichlorophenoxyacetic acid, 23.8% [280 g l^-1^], plus bromoxynil octanoate, 23.8% [280 g l^-1^]), mixed with Axial (pinoxaden, 5.05% [50 g l^-1^], plus cloquintocet-mexyl, 1.26% [12.5 g l^-1^]).

Each year, the central six rows of each plot were combine harvested after physiological maturity. For the collection, this occurred on June 22, 2004, June 15, 2005, and June 21, 2006. For the breeding population, this occurred on June 23, 2013, July 1, 2014, and June 15, 2015. A plot combine harvester was used for threshing and to obtain the grain yield for each plot.

### Agronomic Traits

Heading time (HT; days) was recorded as the number of days from April 1 until the ears of 50% of the tillers had emerged from the flag-leaf sheaths by approximately half of their length (i.e., Zadoks scale, growth stage 55; Zadoks et al., 1974). Plant height (PH; cm) was measured from the ground to the tip of the ear (excluding awns), during the early dough development stage (i.e., Zadoks scale, growth stage 83), when the maximum height was achieved, on five main culms per plot. Grain yield (GY; t ha^-1^) was determined and expressed at 13% moisture level. The grain was stored in a cool chamber at 5°C until analysis. Thousand-grain weight (TGW; g) was calculated as the mean weight of five sets of 200 grains per plot. Specific weight (SW; kg hl^-1^), an indication of the density of the grain, was measured on 250-g samples per plot, using a Schopper Chondrometer equipped with a 1-L container, without reference to the moisture content. Protein content (PC; %N ×5.70) and gluten content (GC; %) were determined on samples of 500 g kernels, using a near-infrared spectrometer (Infratec 1241 Grain Analyser; Foss Electric Analytical A/S, Hillerød, Denmark). For the breeding population, during the vegetative period, the susceptibilities against four fungal diseases were evaluated, according to a 1 to 9 scale (susceptibility index, SI), where 1 represented low performance for a trait; i.e., no disease susceptibility. These fungal diseases were: powdery mildew (*Erysiphe graminis f.sp. tritici* Em. Marchal; PM); septoria leaf spot (*Septoria tritici* blotch; STB); leaf brown rust (*Puccinia recondita* Rob. ex Desm. f. sp. *Tritici*; LR); and yellow rust (*Puccinia striiformis* West; YR).

### Statistical Analysis

For the collection experiment, descriptive statistics include mean, standard error (SE), range (min-max), and coefficient of variation (CV, %), which were calculated over the three growing seasons to describe the variability among the Khorasan accessions. The mean and standard deviation (SD) calculated for seven quantitative traits were used to group the accessions into five classes according to Shakhatreh et al. (2010): ≤mean - 2SD; ≤mean - SD; > mean +/- SD; ≥mean + SD; ≥mean + 2SD. The phenotypic frequency data of each trait were analyzed as the Shannon-Weaver diversity index, *H′*, as shown in Equation (1) and suggested by Grenier et al. (2000):

(1) $$H^{\prime }=-\sum_{i=1}^{n}p_{i}\log_{e}p_{i}$$

where *p*_i_ is the proportion of the entries in the i^th^ class of an *n*-class trait, *n* is the number of the phenotypic classes for a trait, *p*_i_ is the relative frequency, and log_e_ is the natural logarithm. Each value of *H′* was divided by its maximum value, log_e_ *n*, and normalised, to keep the values between 0.0 (monomorphic trait; i.e., all individuals belong to one and the same category) and 1.0 (maximum diversity; i.e., individuals are equally dispersed among the *n* classes). *H′* was estimated on the whole dataset $H_{t}^{\prime }$, then the proportion of the diversity present was partitioned as within the four regions (Africa, Asia, Europe, others) ($H_{\text{s}}^{\prime }=H_{\text{r}}^{\prime }/H_{\text{t}}^{\prime }$, where $H_{\text{r}}^{\prime }=1/n\sum H_{\text{t}}^{\prime }$ is the average diversity over the regions) and between the four regions $\left[{\text{G}}_{\text{st}}=\left(H_{\text{t}}^{\prime }-H_{\text{r}}^{\prime }\right)/H_{\text{t}}^{\prime }\right]$.

Analysis of variance (ANOVA) was performed for each trait according to GLM procedure, using JMP software (version 8.0; SAS Institute Inc., Cary, NC, USA). Homogeneity of the error variance was verified using Bartlett’s tests. Mean comparison was performed by applying the Tukey-Kramer tests, with statistically significant differences determined at the probability level of *P* ≤0.05.

In the breeding population, the total phenotypic variation of each trait was partitioned into the variance components due to genetic and nongenetic factors, according to Falconer and Mackay (1996). Genotypic variance ($\sigma_{\text{g}}^{2}$), phenotypic variance ($\sigma_{\text{p}}^{2}$), and coefficients of variation of the genotypes (GCV) and phenotypes (PCV) were estimated.

Broad-sense heritability ($h_{\text{b}}^{2}$) was estimated as:

(2) $$h_{b}^{2}=\sigma_{g}^{2}/\sigma_{p}^{2}\times 100$$

where $\sigma_{\text{g}}^{2}$ is the genotypic variance and $\sigma_{\text{p}}^{2}$ is the phenotypic variance. The components of the phenotypic variance were calculated on the basis of the analysis of variance of the expected mean squares. The phenotypic variance was therefore calculated as follows:

(3) $$\sigma_{p}^{2}=\sigma_{g}^{2}+\sigma_{\;\mathrm{gy}}^{2}/y+\sigma_{\;e}^{2}/r\times y$$

where $\sigma_{\;\mathrm{gy}}^{2}$ is the genotype × year interaction variance, $\sigma_{\;e}^{2}$ is the error variance, r is the number of replicates, y is the number of years. According to Crespel et al. (2014), years were considered as different environments.

Response to selection (R) was calculated as:

(4) $$R=h_{\;b}^{2}\times i\times \surd \sigma_{\;p}^{2}$$

where, i is the standardized selection differential at 5% selection intensity (i = 2.063).

Then, the R as a percentage of the mean (ΔG) was calculated to compare the extent of predicted R of the different traits under selection, using the following formula:

(5) ∆G (%) = R/μ × 100

The phenotypic (r_p_) and genotypic (r_g_) correlation coefficients for the different traits for all possible combinations were estimated using variances and covariances, according to the method described by Sharma (1998):

(6) $$r_{p}=\;{\mathrm{cov}}_{p1-2}/\surd \left(\sigma_{\;p1}^{2}\times \;\sigma_{\;p2}^{2}\right)$$

(7) $$r_{g}=\;{\mathrm{cov}}_{g1-2}/\surd \left(\sigma_{\;g1}^{2}\times \;\sigma_{\;g2}^{2}\right)$$

where cov_p1-2_ and cov_g1-2_ are the phenotypic and genotypic covariances between traits 1 and 2, and $\sigma_{p1}^{2}$, $\sigma_{p2}^{2}$ and $\sigma_{g1}^{2}$, $\sigma_{g2}^{2}$ are the phenotypic and genotypic variances, respectively, of the two traits.

Contributions of different traits to multivariate polymorphism were tested by PCA. According to Jolliffe (2002), to better understand the relationships and similarities and dissimilarities, and to identify the best genotype, hierarchical clustering were used. PCA was calculated to extract the factorial load of the matrix and also to estimate the number of factors. Principal components with an eigenvalue ≥1 were retained for analysis and subjected to hierarchical cluster analysis. Cluster analysis was performed to measure the hierarchical similarity among the genotypes.

A Euclidean distance matrix was established from the PCA values, to obtain a relative dendrogram (Kamara et al., 2003; Salihu et al., 2006). The entries were clustered using Ward’s minimum variance method. To identify the group to which each genotype belonged, the automatic truncation available as part of the software was used. The means and standard deviations of traits for clusters were obtained from cluster analysis. To determine the significant differences of each trait among the clusters, nested analysis of variance (NANOVA) was performed using the restricted maximum likelihood procedure.

The statistical analyses were performed using two software programs: the JMP software (version 8.0; SAS Institute Inc., Cary, NC, USA); and the R package Agricolae (version 1.2-1) (http://cran.r-project.org/web/packages/agricolae/index.html).

## Results

### Collection Population

The descriptive statistics and the ANOVA results for the recorded phenotypic traits of the 77 Khorasan accessions of the collection population are summarised in **Table 2**. Significant differences (P ≤0.01, P ≤0.001) and a wide range of variation were observed for all of the traits. Grain yield ranged from 1.0 to 4.5 t ha^-1^, with an overall mean of 2.8 t ha^-1^; furthermore, some genotypes showed high values for very important agronomic and qualitative traits, such as: SW (84.5 kg hl^-1^), TGW (70.9 g), PC (15.9%), and GC (12.2%). HT was 25.0 days to 39.0 days, and PH was 90.0 cm to 125.0 cm. The widest range was recorded for GY (CV = 21.3%), followed by TGW (CV = 13.1%), with the narrowest range for SW (CV = 4.8%). Broad-sense heritability (h^2^_b_) for the whole collection was high (>85%) for all traits except for GY (29.2%).

**Table 2:** Mean, standard error (SE), range, coefficient of variation and broad-sense heritability (h^2^_b_) for the seven agronomic traits of the 77 Khorasan accessions grown at Foggia (Italy) over the three growing seasons (from 2005 to 2007).

Trait	Mean ± SE	Range	Coefficient of variation (%)	h^2^_b_ (%)
Heading time (days)^†^	33.0 ± 0.2	25.0–39.0***	6.9	92.62
Plant height (cm)	109.3 ± 0.5	9.0–125.0***	7.3	85.37
Grain yield (t ha^-1^)	2.8 ± 0.1	1.0–4.5**	21.3	29.20
Specific weight (kg hl^-1^)	77.7 ± 0.2	68.2–84.5**	4.8	97.68
1000-grain weight (g)	54.0 ± 0.5	37.1–70.9**	13.1	99.14
Protein content (%)	13.4 ± 0.1	10.5–15.9***	5.4	85.35
Gluten content (%)	10.2 ± 0.1	9.0–12.2***	6.6	87.98

^†^From April 1.

**, *** Statistically significant: 1%, 1‰ level of probability, respectively.

The $H_{t}^{\prime }$ estimate based on the frequencies of the entire dataset was 0.55, and the traits differed in the capacity to detect total diversity that ranged from 0.39 for PC, to 0.67 for TGW, followed by SW (0.61), GC (0.57), PH (0.57), and GY (0.55) (**Table 3**). Total gene diversity $H_{t}^{\prime }$ of a germplasm collection was partitioned into the mean gene diversity within geographic regions ($H_{s}^{\prime }$) and the gene diversity between regions ($G_{\mathrm{st}}$). Partitioning of the phenotypic diversity showed that 87% of the total variation was within the regions of origin, while only 13% was between the regions. Among the seven traits, GY, PC, and GC showed high within region variation ($H_{\text{ s}}^{\prime }=92\%,90\%,92\%$, respectively), whereas, in decreasing order, TGW, PH, and SW contributed relatively more to the regional differentiation (G_st_ = 27, 15, and 14%, respectively); the remaining traits showed much lower G_st_ (8–12%).

**Table 3:** Phenotypic diversity index and partitioning within the four geographic regions of Africa, Asia, Europe, and others, based on the seven traits of the 77 Khorasan accessions.

Trait	Statistic			
	$H_{t}^{\prime }$ ^‡^	$H_{r}^{\prime }$	$H_{s}^{\prime }$	$G_{\mathrm{st}}$
Heading time (days) ^†^	0.51	0.45	0.88	0.12
Plant height (cm)	0.57	0.48	0.85	0.15
Grain yield (t ha^-1^)	0.55	0.50	0.92	0.08
Specific weight (kg hl^-1^)	0.61	0.52	0.86	0.14
1000-grain weight (g)	0.67	0.49	0.73	0.27
Protein content (%)	0.39	0.35	0.90	0.10
Gluten content (%)	0.57	0.53	0.92	0.08
**Means**	**0.55**	**0.48**	**0.87**	**0.13**

^†^From April 1.

^‡^$H_{t}^{\prime }$_t_ total diversity; $H_{r}^{\prime }$, average diversity over regions; $H_{s}^{\prime }$, diversity within regions; $G_{\mathrm{st}}$ level of differentiation between regions.

A heat map was constructed to visualize the traits that had weak (r ≤0.21), moderate (r = 0.22–0.27) and strong (r = 0.28–1.00) correlations (**Supplementary Figure S1**). This analysis showed that 43% of the trait combinations had strong correlations, while 10% had moderate correlations. The highest correlation coefficients were between HT and PH (r = 0.53, P ≤0.01), SW and TGW (r = 0.68, P ≤ 0.01), GY and SW (r = 0.51, P ≤0.01), and PC and GC (r = 0.66, P ≤ 0.01). GY also correlated with TGW, PC, and GC (0.32, 0.32, 0.33, P ≤ 0.01, respectively).

The structure of the genetic diversity among the accessions was examined with PCA on the seven traits. The first three axes showed eigenvalues >1.0 and explained ∼78% of the observed phenotypic diversity for the whole Khorasan germplasm collection (**Supplementary Table S2**). The first and second axes accounted for 35.8% and 27.5% of the total variation, respectively. Three traits, SW, TGW, and GY (in decreasing order), showed positive eigenvector loading, and explained 57% of the total variation in the first principal component, whereas HT, protein content, and PH (in decreasing order) explained 61% of the total variation in the second principal component. The third principal component explained 14.7% of the variance, and was associated with gluten content and HT.

**Figure 1:**
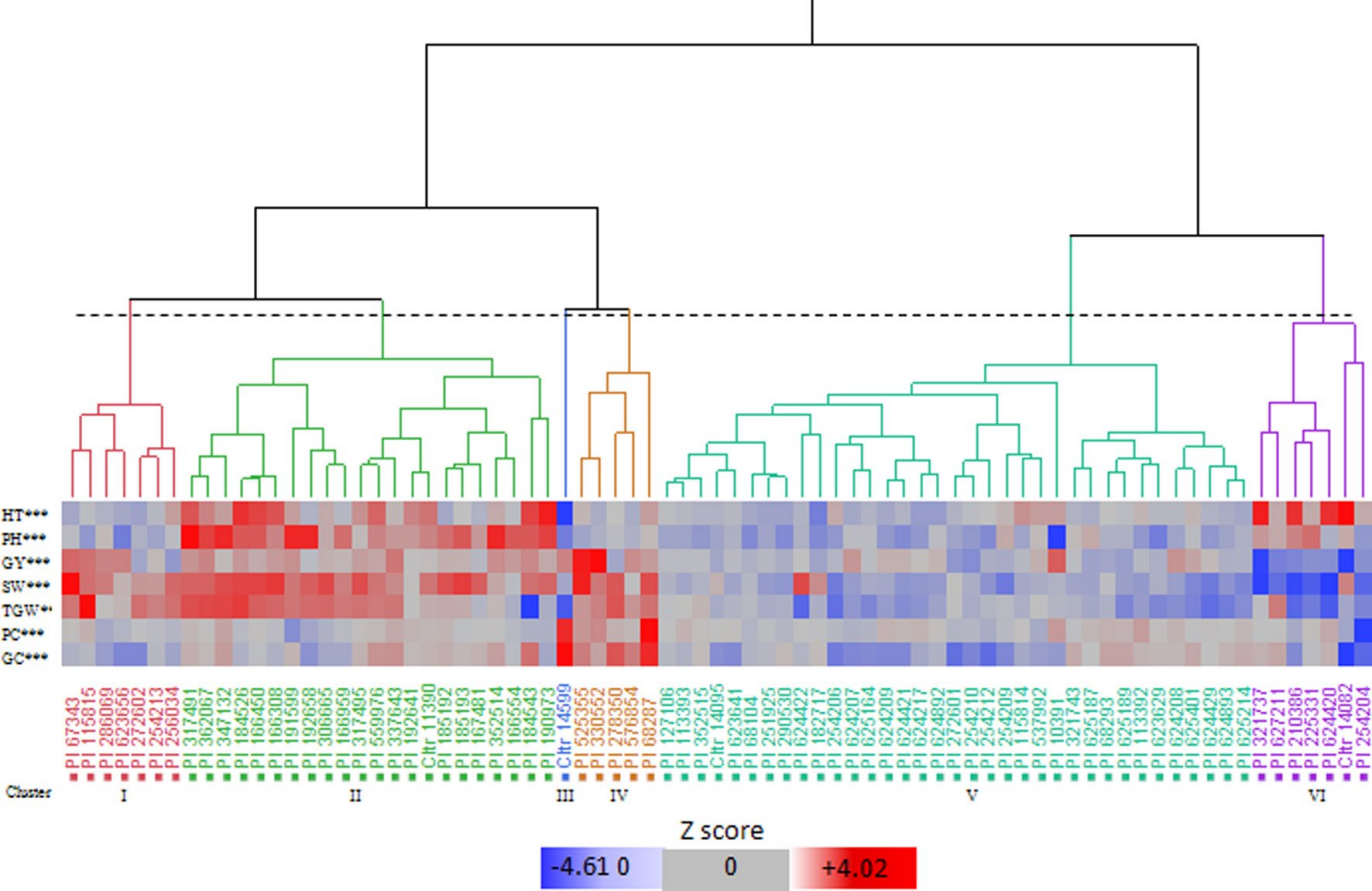
Hierarchical cluster analysis dendrogram (Ward’s method) and pattern map of the values of the bio-agronomic traits for the 77 Khorasan wheat genotypes. HT, heading time; PH, plant height; GY, grain yield; SW, specific weight; TGW, 1000-grain weight; PC, protein content; GC, gluten content. Dotted line, 20% threshold point delineating six clusters. ***, significant differences at 0.001 level among clusters (Tukey-Kramer test).

The cluster analysis identified six clusters, i.e. I to VI, which included 7, 22, 1, 5, 35, and 7 accessions, respectively (**Figure 1**; **Supplementary Table S3**). Based on the means calculated over the genotypes included in each cluster (**Supplementary Table S3**), we found that cluster I showed high TGW, and cluster II was characterised by the highest PH and SW. Cluster III had a single accession, which was distinct from each of the others in terms of its high protein content and gluten content and its low HT and PH. Cluster IV had the highest GY, SW, and TGW, and good protein and gluten contents. Cluster V had intermediate values for almost all of the traits, and finally, cluster VI included the genotypes with high PH and the latest HT.

According to the Shannon index, despite ecological and geographic differences within each cluster, there were similar accessions from regions that were distant from each other (**Figure 1**). However, this classification suggested that most of the accessions from Iran and adjacent regions had a tendency to come together in cluster V. The dendrogram in **Figure 1** shows the similarities of the accessions examined. The pattern map of the traits for each genotype allowed the identification of seven genotypes with desirable variants of each trait for the use as gene donors for the RIL breeding populations: PI67343, PI525355, PI330552, PI10391, PI68287, CItr14599, and PI115815. Based on the results of the multi-year trials, the selected genotypes showed phenotypic stability and combined high grain yield and/or protein and gluten contents, and good agronomic performance (**Supplementary Table S4**). In particular, all these genotypes had high GY potential (> mean + 0.58 SD) and other favourable traits, such as: high SW (>mean + 3.64 SD), high TGW (>mean + 7.00 SD), high protein content (>mean + 0.56 SD), high gluten content (>mean + 0.53 SD), low HT (<mean - 1.87 SD), low PH (<mean - 6.50 SD). In particular, across these accessions, PI67343 and PI115815 (cluster I) had high SW and TGW, CItr14599 (cluster III) had high qualitative traits of protein and gluten contents, PI68287, PI525355, and PI330552 (cluster IV) had high SW, TGW, protein content, and gluten content, and PI10391 (cluster V) had the lowest PH.

### RILs Population

Significant differences (P < 0.0001) were seen among the *Genotype* (*G*), *Year* (*Y*), and *G* × *Y* interaction effects for all of the traits examined. Compared to durum wheat, the Khorasan parent accessions had later HT (+10 days), higher PH (+38 cm), lower GY (-2 t ha^-1^), higher SW (+10 kg hl^-1^) and higher protein content (+1%) and gluten content (+0.8%) (**Table 4**). The means across all RILs for SW, TGW, protein, and gluten content traits were higher than those of the durum wheat parents. The data indicated wide ranges of variation and most of the backcross lines were characterized with improved agronomic characters. In particular, the highest GY (6.7 t ha^-1^), SW (67.9 kg hl^-1^), TGW (85 g), protein content (17.9%), and gluten content (14.3%) were seen for the breeding population genotypes. Furthermore, some of the RILs produced significantly early heading and shorter plants. The extent of the variability was assessed by estimation of the genetic parameters (**Table 4**). For all traits, a large portion of the phenotypic variance (σ^2^_p_), i.e. more than 68%, was accounted for by the genetic component (σ^2^_g_). The phenotypic coefficient of variation (PCV) was higher than the genotypic coefficient of variation (GCV) for all of the traits. The PCV and GCV were high (>20%) for GY and SI for diseases, intermediate (10–20%) for HT, PH, and SW, and low (< 10%) for the other traits. Broad-sense heritability for the whole collection was high, and ranged from 68.2% (STB score) to 98.2% (PH). Heritability was >90% also for HT, GY, and TGW. Selection gains of more than 10% would be expected in 10 traits if 5% of the lines were selected. ΔG was particularly high for SI for diseases (>69.4%) and GY (63.7%), while it was minimal for TGW (4.6%). High heritability and genetic gain were recorded for SI for diseases, GY, PH, and HT.

**Table 4:** Phenotypic performance and estimates of genetic parameters of the 11 agronomic and disease-resistance traits in the parent lines and in the breeding population derived from the *T. durum* × *T. turanicum* crossing.

Trait	Durum parental	Khorasan parental	Breeding population	Whole collection								
	Mean ± SE	Mean ± SE	Mean ± SE	Mean	Range	CV^&^(%)	$\sigma_{\;g}^{2}$	$\sigma_{\;p}^{2}$	GCV (%)	PCV (%)	$h_{\;b}^{2}$ (%)	ΔG (%)
Heading time (days)^†^	27.0 ± 1.5	37.0 ± 1.0	29.8 ± 0.1	29.9	21.0–40.7	10.8	10.31	10.73	10.74	10.96	96.09	21.68
Plant height (cm)	76.7 ± 1.0	115.0 ± 2.4	100.0 ± 0.7	100.0	63.3–141.7	19.1	364.13	370.73	19.08	19.25	98.22	38.96
Grain yield (t ha^-1^)	4.2 ± 0.1	2.2 ± 0.2	3.2 ± 0.1	3.2	1.01–6.7	31.3	1.00	1.02	31.25	31.56	98.04	63.74
Specific weight (kg hl^-1^)	40.0 ± 3.1	50.0 ± 2.9	49.6 ± 0.3	49.6	28.0–67.9	15.5	53.89	70.45	14.80	16.92	76.49	26.67
1000-grain weight (g)	79.7 ± 0.9	79.4 ± 0.8	80.6 ± 0.1	80.6	72.2–85.0	2.4	3.57	3.88	2.34	2.44	92.01	4.63
Protein content (%)	14.9 ± 0.5	15.9 ± 0.3	15.7 ± 0.1	15.6	12.5–17.9	6.1	0.86	1.00	5.94	6.41	86.00	11.36
Gluten content (%)	11.1 ± 0.2	11.9 ± 0.3	11.5 ± 0.1	11.5	8.0–14.3	8.1	0.83	0.94	7.92	8.43	88.30	15.33
PM score^‡^	1.2 ± 0.1	4.1 ± 0.7	2.0 ± 0.1	2.0	0.0–6.7	63.6	1.55	1.84	62.25	67.82	84.24	117.70
STB score	1.7 ± 0.4	4.3 ± 0.4	1.9 ± 0.1	1.9	0.3–6.0	43.6	0.60	0.88	40.77	49.37	68.18	69.35
YR score	0.0 ± 0.0	0.0 ± 0.0	0.9 ± 0.1	0.9	0.0–6.3	106.1	0.79	1.05	98.76	113.86	75.24	176.46
LR score	3.1 ± 1.2	1.2 ± 0.3	2.7 ± 0.1	2.7	0.0–7.7	68.8	3.30	3.70	67.28	71.24	89.19	130.89

^†^From April 1.

^‡^PM, powdery mildew resistance; STB, Septoria tritici blotch resistance; YR, yellow rust resistance; LR, brown rust resistance.

^&^CV, coefficient of variation; σ^2^_g_, genotypic variance; σ^2^_p_, phenotypic variance; GCV, genetic coefficient of variation; PCV, phenotypic coefficient of variation; h^2^_b_, broad-sense heritability; ΔG, expected response to selection at 5% of the selection intensity.

Grain yield had significant positive phenotypic correlation with TGW (r_p_ = 0.31, P ≤ 0.05), and significant negative association with HT (r_p_ = -0.34, P ≤ 0.01), PH (r_p_ = -0.20, P ≤ 0.05), YR score (r_p_ = -0.34, P ≤ 0.01), and LR score (r_p_ = -0.40, P ≤ 0.01) (**Table 5**). GY showed a not significant negative association with protein and gluten content. TGW was also positively correlated with PH (r_p_ = 0.40, P ≤ 0.01) and SW (r_p_ = 0.43, P ≤ 0.01), and the association of protein content with gluten content was positive and significant (r_p_ = 0.92, P ≤ 0.01). The genetic correlation coefficients were higher than their corresponding phenotypic ones.

**Table 5:** Phenotypic (above diagonal) and genotypic (below diagonal) coefficients of correlation (*r*) between the 11 agronomic and disease-resistance traits for the 800 genotypes evaluated in 2013–2015.

Trait^†^	HT	PH	GY	SW	TGW	PC	GC	PM	STB	YR	LR
HT		0.07	–0.34**	0.14	0.13	0.07	0.14	0.19	0.02	0.20*	0.08
PH	0.07		–0.20*	0.58**	0.40**	0.20*	0.19	0.02	–0.09	0.03	–0.13
GY	–0.34^++^	–0.20^++^		–0.10	0.31*	–0.09	–0.08	–0.19	0.15	–0.34**	–0.40**
SW	0.15	0.61^++^	–0.10		0.43**	0.15	0.14	–0.05	–0.09	0.08	–0.11
TGW	0.13^+^	0.41^++^	0.31^++^	0.46^++^		0.01	0.04	–0.04	0.02	–0.08	–0.24*
PC	0.07	0.21^++^	–0.09^+^	0.17^+^	0.00		0.92**	0.17	0.08	–0.01	–0.19
GC	0.15^+^	0.20^++^	–0.08^++^	0.15^+^	0.03	0.94^++^		0.17	0.10	0.01	–0.20*
PM score	0.20^+^	0.03	–0.20^++^	–0.05	–0.04	0.17^++^	0.17^++^		0.52**	0.33**	.018
STB score	0.02	–0.10^++^	0.15^++^	–0.10	0.02	0.09^+^	0.11^++^	0.56^++^		0.12	–0.06
YR score	0.22^+^	0.03	–0.35^++^	0.09	–0.08^+^	–0.01	0.01	0.36^++^	0.14^++^		0.31**
LR score	0.08	–0.13^+^	–0.51^++^	–0.12^+^	–0.25^++^	–0.20^++^	–0.21^++^	0.18^++^	–0.06^+^	0.33^++^	

^†^HT, days to heading from April 1; PH, plant height; GY, grain yield; SW, specific weight; TGW, 1000-grain weight; PC, protein content; GC, gluten content; PM, powdery mildew resistance; STB, Septoria tritici blotch resistance; YR, yellow rust resistance; LR, brown rust resistance.

Genotypic coefficients: significance of a genetic coefficient correlation is expressed as being greater than once (+) or twice (++) its SE

*, ** Significance at P < 0.05, P < 0.01, respectively.

Due to the high phenotypic variation, the PCA showed wide dispersion along both of the PC axes. For the RILs population, four principal components had eigenvalues >1 and accounted for 70% of the total variance (**Supplementary Table S5**). The proportions of the total variance attributable to the first four principal components were 22.0, 19.6, 15.7, and 12.8%. PH, protein content, and gluten content had the highest loadings for the first principal component, which indicates that they contributed significantly to this component. GY, PM score, YR score, and LR score contributed to the second principal component. SW was the main trait that contributed to the third principal component, and for the fourth principal component, TGW, PM score, and STB score were most important.

Cluster analysis was performed considering all measured traits, and this divided the 800 genotypes into 20 major clusters, I to XX (**Table 6**). Nested ANOVA revealed significant differences between these clusters for all traits. Based on the mean performance of each trait, cluster IV was composed of early HT (-25.1 days) and high GY (+4.1 t ha^-1^) genotypes. Clusters VIII and XIV were composed of genotypes with good qualitative traits, i.e. protein content (+16.9%, on average) and gluten content (+12.6%, on average). Cluster IX comprised genotypes that were characterized by high PH (+116.0 cm). Genotypes that constituted clusters X and XII showed higher PH (+115.7 cm, on average), higher SW (+82.0 g) and lower SI for diseases (-1.8, on average). Cluster XIII comprised genotypes that were characterized by late HT (+34.6 days). Cluster XVIII was composed of genotypes with high GY (+4.1 t ha^-1^) and low SI for diseases (-1.3, on average). Finally, early HT (-28.4 days), short PH (-78.9 cm) and high GY (+4.7 t ha^-1^) were the special features of cluster XX.

**Table 6:** Mean values for the 11 agronomic and disease-resistance traits according to the 20 clusters grouped from the cluster analysis of the 800 genotypes derived from the *T. durum* × *T. turanicum* crossing.

Cluster	No. of genotypes	Agronomic trait^†^							Disease-resistance trait^†^			
		HT (days)	PH (cm)	GY (t ha^-1^)	SW (kg hl^-1^)	TGW (g)	PC (%)	GC (%)	PM score	STB score	YR score	LR score
I	33	31.9 b	77.5 j	1.7 m	41.7 jk	76.7 l	15.3 fg	11.3 e–h	1.8 e–g	1.3 hi	1.3 cd	**5.4 a**
II	34	32.3 b	78.9 j	2.5 ij	46.6 i	79.5 i	15.5 d-f	11.6 d–f	1.2 i	1.4 g–i	1.1 de	2.7 f
III	49	29.5 de	78.9 j	3.2 e	40.9 k	79.1 j	15.7 de	11.5 e–g	2.3 d	2.0 ef	0.8 gh	4.8 bc
IV	26	25.1 g	111.2 c	4.1 b	51.1 d-f	81.8 b	14.4 j	10.4 j	1.2 i	1.7 fg	0.5 jk	1.8 h
V	29	28.9 de	84.5 i	3.9 c	47.0 hi	81.5 bc	13.7 k	9.8 k	1.3 hi	2.1 de	0.4 j–l	2.4 fg
VI	16	28.7 de	94.6 g	2.3 k	47.5 g–i	80.3 e–g	13.6 k	9.5 k	1.2 i	1.5 gh	1.2 c–f	5.3 ab
VII	39	26.2 fg	83.1 i	3.6 d	42.9 j	78.5 k	14.8 i	10.5 j	1.2 i	1.5 gh	0.5 jk	2.7 f
VIII	41	29.3 de	111.5 c	2.6 i	50.0 e–g	79.7 hi	**16.8 a**	**12.6 a**	1.7 gh	1.4 g–i	0.8 g–i	1.8 h
IX	48	25.9 g	**116.0 a**	3.0 f	55.4 bc	80.4 ef	16.2 c	11.9 d	1.3 i	1.4 g–i	0.6 h–j	2.6 fg
X	83	32.4 b	**115.6 a**	2.8 g	54.6 c	81.8 b	15.7 d	11.6 de	1.3 i	1.2 i	0.9 fg	2.3 g
XI	60	31.6 b	115.2 ab	2.8 g	56.5 ab	81.2 c	15.0 hi	10.9 i	2.0 e	1.6 g	1.0 e–g	4.2 d
XII	49	31.6 b	**115.8 a**	2.7 h	**57.3 a**	**82.2 a**	16.5 ab	12.5 ab	2.7 c	2.2 de	1.4 c	2.4 fg
XIII	17	**34.6 a**	112.8 bc	2.9 g	52.0 de	81.4 bc	16.2 bc	12.3 a–c	3.7 b	**3.6 a**	1.1 d–g	1.5 hi
XIV	17	28.1 ef	108.3 d	2.8 gh	50.1 d–g	80.6 de	**16.9 a**	12.6 ab	3.8 b	2.5 cd	1.2 c–f	5.0 ab
XV	16	31.8 bc	98.1 f	2.4 j	48.2 g–i	79.8 g–i	15.4 d–h	11.4 e–h	3.9 b	3.0 b	**4.9 a**	4.4 cd
XVI	24	32.0 b	105.3 e	2.1 l	48.9 f–h	80.0 f–h	15.5 d–g	11.2 gh	**5.3 a**	2.8 bc	1.8 b	3.7 e
XVII	26	29.4 de	100.9 f	3.3 e	45.8 i	81.7 b	15.2 g–i	11.1 hi	2.8 c	2.8 bc	1.2 c–e	3.5 e
XVIII	89	29.9 cd	105.8 e	4.1 b	52.3 d	81.8 b	16.2 c	12.2 bc	1.9 ef	2.2 de	0.2 l	0.9 j
XIX	45	29.1 de	87.4 h	3.6 d	41.2 jk	79.7 hi	16.2 bc	12.2 c	2.5 cd	2.1 de	0.6 ij	1.5 hi
XX	59	28.4 e	78.9 j	**4.7 a**	46.9 hi	80.8 d	15.5 e–g	11.3 f–h	1.8 fg	2.3 de	0.3 kl	1.3 i

^†^HT, days to heading from April 1; PH, plant height; GY, grain yield; SW, specific weight; TGW, 1000-grain weight; PC, protein content; GC, gluten content; PM, powdery mildew resistance; STB, Septoria tritici blotch resistance; YR, yellow rust resistance; LR, brown rust resistance.

Data with different letters within columns indicate significant differences (Tukey-Kramer tests).

Data in bold text indicate highest value(s) within each column (i.e., trait) across the 20 clusters.

**Table 7** presents the number of superior genotypes for each trait within each group based on the genotypes with trait values that exceeded the mean plus SD and with a high probability of success in the selection where there is sufficiently high heritability. For HT, PH, and SI for diseases, mean minus SD was used, as these traits are useful for better crop performance under Mediterranean conditions. According to these criteria, the highest number of superior genotypes was 205 for PH and 206 for YR score, and the lowest number was 110 for protein content and 124 for gluten content. Six of the lines showed better performance for at least six different traits. These superior lines showed increased GY (+64.5%), SW (+28.5%), TGW (+3.5%), protein content (+6.4%), and gluten content (+6.8%), and decreased HT (-24.9%), PH (-20.8%), and pathogen susceptibility (-79.2%, on average). Compared to the parents, line 721 was 8 days earlier for HT, and the three lines 630, 721, and 754 were 20 cm shorter, on average, for PH, and 65% more productive, on average, as GY. In particular, line 696 was superior according to seven of the traits.

**Table 7:** Number and means of genotypes with superior values for each of the 11 agronomic and disease-resistance traits, and superior lines for multiple traits, as identified by cluster analysis, with comparisons to parent values also given.

Trait	No. of genotypes	Mean	Superior line for multiple traits							Parent comparison	
			382 (X)^&^	422 (XVIII)	630 (XVIII)	696 (XVIII)	721 (XX)	754 (XVIII)	Mean* (M)	Parent mean (PM)	Difference (%)
Heading time (days) ^†^	129	24.9	–	–	–	–	24.7	–	24.7	32.9	–24.9
Plant height (cm)	205	74.4	–	–	73.3	–	78.3	78.3	76.6	96.7	–20.8
Grain yield (t ha^-1^)	124	4.9	–	–	4.7	–	5.2	5.5	5.1	3.1	+64.5
Specific weight (kg hl^-1^)	152	60.2	57.5	–	–	62.3	–	–	59.9	46.6	+28.5
1000-grain weight (g)	132	83.1	82.8	83.2	–	–	–	83.4	83.1	80.3	+3.5
Protein content (%)	110	17.0	16.7	16.6	16.6	16.7	–	17.0	16.7	15.7	+6.4
Gluten content (%)	124	12.8	12.6	12.6	12.5	12.5	12.5	12.7	12.6	11.8	+6.8
PM score^‡^	143	0.62	0.67	0.67	–	0.67	–	–	0.67	3.28	–79.6
STB score	176	0.96	1.00	1.00	–	1.00	–	–	1.00	3.06	–67.3
YR score	206	0.00	–	–	0.00	0.00	0.00	–	0.00	0.00	–0.00
LR score	129	0.32	–	0.00	0.00	0.33	0.67	0.00	0.20	2.17	–90.8

^†^From April 1.

^‡^PM, powdery mildew resistance; STB, Septoria tritici blotch resistance; YR, yellow rust resistance; LR, brown rust resistance.

^&^Corresponding cluster.

*M, mean of the lines with superior traits; PM, mean of 7 Khorasan accessions and 3 durum wheat cultivars; Difference (%) = [(M - PM)/PM×100]

Superior trait for HT, PH, PM, STB, YR, and LR: mean - σ; for GY, SW, TGW, PC and GC: mean + σ.

## Discussion

Phenotypic variation in Khorasan wheat has not been studied in great detail. However, knowledge of the extent of variability for plant traits and the association of specific traits within the collected material would help to revisit the breeding programs (Mengistu et al., 2016b).The data showed significant variations among the Khorasan accessions particularly for GY and TGW, which can be used to develop higher yielding cultivars through breeding. Grausgruber et al. (2005) reported average yields of the best performing Khorasan wheats of about 440 g m^-2^ for the autumn crop grown in Central Europe. These values are comparable to the best accessions evaluated in the present study, in which we adopted a similar sowing density (300–350 seeds m^-2^). Also, the means observed in the present study for SW (77.7 kg hl^-1^) and TGW (54.0 g) were similar to those reported by Grausgruber et al. (2005) and Sissons and Hare (2002), for the environmental conditions of Central Europe and Australia, respectively. The size and shape of the wheat grain can have significant effects on grain weight and flour yield. Here, the particularly high TGW is explained by the very large kernel size, which is up to twice the size of durum wheat kernels. The narrow and flinty kernel shape, which reduces the density of agglomeration within the chondrometer, is the reason for a relatively low hectoliter weight (Stagnari et al., 2008).

Protein content represents an important factor for price determination in wheat trading, and semolina PC >12% is considered one of the parameters required to obtain good pasta quality (Ross and Bettge, 2009). According to Piergiovanni (2009), experimental evidence suggests that Khorasan showed a tendency to accumulate high protein content. Similar to our data, Stagnari et al. (2008) reported protein content and gluten content of 16.1 and 12.1%, respectively, in 13 lines of Khorasan evaluated in southern Italy.

The estimate of heritability provides index of transmissibility of characters. In our Khorasan collection, the heritability value for GY was low, suggesting that selection for this single character would not be effective due to predominant effects of non-additive genes. The phenotypic diversity of the gene pool of Khorasan wheat is reflected on the *H*′_t_ estimates. According to Eticha et al. (2005), the phenotypic frequencies for each trait analyzed by the Shannon-Weaver diversity index can be classified as high (*H*′ ≥ 0.60), intermediate (0.40 ≤ *H*′ ≤ 0.60), and low (0.10 ≤ *H*′ ≤ 0.40). The overall genetic diversity (*H’*_t_) was medium to high (0.55) in the present study, which indicates that the Khorasan wheat accessions had a substantial level of genetic diversity, with relatively high frequency for the desirable classes of many of the agronomically important traits. While most traits showed relatively high levels of polymorphism, only protein content had low *H’*_t_ estimates which might reflect unequal frequencies of different classes rather than the absence of the desirable class for a particular trait (Jaradat and Shahid, 2006). Furthermore, our data indicate that most of the variation between accessions is due to differences within regions, rather than across different regions. According to Bekele (1984), it appears that collection strategies depend very much on variations in the ecologic-climatic and/or topographic conditions within a collection site. In areas with significant environmental variation, a larger number of collection sites should be considered.

Using Pearson’s correlations, we determined the traits with moderate to high correlations that can be considered by breeders as important targets during selection (Mwadzingeni et al., 2016). Thus, SW by its positive association with GY, can be used as criteria for indirect selection for productive Khorasan cultivars. Also TGW, protein content, and gluten content showed a moderate positive phenotypic association with GY. Several studies showed inverse relationship between grain yield and grain N concentration (Triboï et al., 2006; Amiri et al., 2018). They suggested that this inverse relationship is because the genetic variations in C assimilation are higher than those of N assimilation. The variability is induced by both genetic and environment factors and their interactions. A positive association between GY and protein content was reported for durum wheat cultivars grown in a Mediterranean environment under organic farming system (Iannucci and Codianni, 2016). These data were confirmed by the PCA analysis, in which the agronomic traits explained a high percentage of the total variation among the accessions. Indeed, HT, PH, SW, and TGW were positive for both the first and second principal components, which indicated that these traits contributed maximally toward divergence, and can be considered as key traits for the estimation of genetic diversity in Khorasan wheat.

The promising genotypes can be identified from the cluster means recorded for each trait. The distribution of the accessions within clusters has no apparent relationship in terms of geographic origin, although many of the Western Asia accessions had a tendency to remain together. The broad diversity of the Khorasan accessions within large regions can be due to different reasons, i.e., the variability of climatic and agricultural conditions, the selection pressure, and the high levels of gene migration (i.e., gene flow) (Jaradat and Shahid, 2006), or it is also possible that classification by regions masks divergence, because different phyto-geographic regions can be included in the same political boundaries (Khan et al., 2009). On the other hand, accessions from very distant regions (i.e., Australia, Turkey, and Spain) may have similar genetic background (i.e., cluster I). These results could be attributed to free exchange of materials that may have overlapped the previous diversity distribution pattern of the domesticated species (Jaradat and Shahid, 2006). Therefore, geographic diversity should not necessarily be used as an index of gene-specific populations. We found five real clusters and a mono-genotype cluster. Interestingly, CItr 14599 (originating from Ethiopia), which is the only genotype that belongs to cluster III, was earliest and had a higher grain yield, which makes it desirable, as this combines two important, but mostly contradicting, traits. Indeed, it has been recently indicated that the geographic area of Ethiopia was a separate domestication center for tetraploid wheats (Mengistu et al., 2016b). The distinctiveness of the Ethiopian materials from any of the other gene pools probably results from geographic barriers that prevented gene flow, or alternatively from intensive selection for adaptive gene complexes (Lopes et al., 2015).

To the best of our knowledge, the present study is the first to evaluate variability of agronomic and qualitative traits in a wide collection of Khorasan wheat under field conditions. These data show a great genetic potential, and the selection of the best genotypes from the clusters with the best combinations of yield-related traits for direct use as cultivars is feasible for Khorasan wheat. Based on univariate and multivariate assessments, seven promising accessions that showed desirable expression for many traits simultaneously were identified as donors for durum wheat improvement. In particular, we identified: PI68287 from Azerbaijan (with high GY, SW, TGW, PC, and GC), PI67343 from Australia and PI115815 from Turkey (with high GY, SW, and TGW), PI525355 from Morocco (with high GY, SW, PC, and GC), PI330552 from United Kingdom (with high GY and PC), PI10391 from Egypt (with high GY and low PH), and CItr14599 from Ethiopia (with high GY, PC, GC and low HT, PH).

The highly significant genotypic differences observed among all of the traits investigated indicate that the germplasm derived from *T. durum* × *T. turanicum* crossing is a rich source of genetic diversity for breeding purposes. High variation was observed here for the SI for diseases, GY, PH, SW, and HT traits. Appreciable variability for several durum wheat traits was reported by Mengistu et al. (2016a), which included HT, PH, aboveground biomass, GY, and other yield components. As reported by Grausgruber et al. (2005), in general, Khorasan wheats have turned out to be medium to very susceptible to the prevalent races of PM, LR and YR. High susceptibility to YR has already been reported by Vavilov (1951). In the present study, low to medium susceptibilities to leaf diseases were observed, probably because of the low rainfall (132 mm) and humidity during the spring period (April-June), which represented 26% of the annual total rainfall recorded for southern Italy (average from 2003–2017).

Information on the magnitude and significance of the genotypic and environmental components of the phenotypic variation for important traits provides a basis for the development of efficient breeding programs (Allard, 1960). In our research, the phenotypic variance (σ^2^_p_) and PCV were a little higher than the corresponding genotypic variance (σ^2^_g_) and GCV for all of the traits, which suggested the presence of environmental influence to some extent in the expression of these traits (Johnson et al., 1955; Ehdaie and Waines, 1989). This was confirmed by the significant *genotype* × *environment* interactions. However, high ratios of genotypic variance to phenotypic variance indicate that there is inherent variability among the genotypes that remains unaltered by environmental conditions, and which in turn, is more useful for exploitation in selection and hybridization programs (Falconer and Mackay, 1996). Here, PCV and GCV were categorized as low (0–10%), moderate (10–20%) and high (> 20%) (Johnson et al., 1955). Accordingly, high PCV and GCV were recorded for SI for diseases and GY, which suggests the possibility of improving these traits through selection based on phenotypic expression. In contrast, TGW, protein and gluten content showed low PCV and GCV. Similar findings were reported by Chatzav et al. (2010) for durum wheat genotypes.

Nevertheless, the proportion of genetic variability that is transmitted from parents to all offspring is reflected by the heritability, which gives information on the relative values of the phenotypic selection. According to Johnson et al. (1955), heritability >80% is very high, from 60–79% is moderately high, from 40–59% is medium, and <40% is low. As a consequence, the particularly high heritability seen here for HT, PH, GY, and TGW (> 90%) shows that these traits are governed by additive genes. Comparable data were reported for durum wheat by Mengistu et al. (2016b). The high heritability obtained might be due to favorable growing conditions and the adaptability of the plant material used. There were high h^2^_b_ values for protein content and gluten content (86 and 88%, respectively), which suggests that these traits are controlled by the genotype more than by the environment, in comparison with SW. Similar data were reported by Zanetti et al. (2001) for *Triticum aestivum* RILs and improved lines.

Falconer and Mackay (1996) stated that heritability estimates together with R are more important than heritability alone for prediction of the effects of selection of the best individuals. R indicates the degree of gain in a trait obtained under one cycle of selection at a given selection intensity, and it helps in the understanding of the type of gene action involved in the expression of various polygenic traits (Allard, 1960). According to Johnson et al. (1955), R can be classified as low (< 10%), moderate (10–20%) and high (> 20%). High and low R are indicative of additive or non-additive gene actions, respectively. The maximum expected R, which was expressed as percentage of the mean (ΔG), was seen for SI for diseases and GY. The high h^2^_b_ and ΔG for GY, PH, HT, SW, and SI for diseases indicates that selection for these traits would be more effective. High h^2^_b_ coupled with moderate ΔG were reported for protein content and gluten content, which indicated the presence of intra-allelic and inter-allelic interactions in the expression of these traits (Falconer and Mackay, 1996). High h^2^_b_ estimates and low expected ΔG were seen for TGW, which indicate the dominant and epistatic nature of inheritance (i.e., nonadditive gene action). The lower R for TGW, protein content, and gluten content despite high heritability estimates are due to low variability. This shows the importance of genetic variability for improvements through selection.

Genotypic and phenotypic correlation analyses among the traits is needed to understand correlated inheritance (Lopes et al., 2015). Between all of the trait combinations, the r_g_ values were close, and a little higher than their corresponding phenotypic values, which might be due to masking or modifying effects of the environment on the genetic association among the traits (Johnson et al., 1955). The negative and significant phenotypic and genotypic association between GY and HT and PH indicates the possibility of identifying lines with a combination of high grain production, early heading, and short plants. These associations might be due to pleiotropy or linkage disequilibrium (Chen and Lübberstedt, 2010), and they have significant importance in durum wheat breeding programs. As De Vita et al. (2007) and González-Ribot et al. (2017) reported, selection for early flowering has been positive for grain yield as this avoids terminal drought stress. So, HT might be used as an indirect selection criterion for better grain yield. Also TGW, an important component of GY, showed significant positive phenotypic and genotypic associations with GY, which implies that improving this trait might provide high GY.

Based on across-year analyses, HT, PH, and TGW are the most promising traits for indirect selection. Similar data were obtained by Mohammadi et al. (2018) for 25 durum wheat breeding genotypes under drought conditions, and by González-Ribot et al. (2017) for a durum wheat collection. The negative relationship between GY and PC and GC might be due to opposite pleiotropic gene effects, caused by the major bio-energetic requirements for synthesis of protein and then carbohydrate (Blanco et al., 2006). This suggests that it is difficult to combine higher GY, protein content, and gluten content in a single genotype.

A high percentage of total variation (70%) was accumulated by the first four principal components, which was explained by the bio-agronomic traits studied. On the basis of the cumulative effects of the individual traits examined, the genotypes were clustered into 20 groups, each of which included 16 to 89 genotypes. The genotypes in each cluster have defined traits, and thus represent resources for specific breeding programs. The results indicated that based on each trait, many RILs from the complex crossing program exceeded the parents (from 110 to 205 genotypes for protein content and PH, respectively). The best breeding lines showed low increments for TGW. Accordingly, De Vita et al. (2007) and Royo et al. (2014) reported that the response to selection of durum wheat in Mediterranean environments is more associated with the number of spikes m^-2^ and kernels m^-2^, than with TGW.

Grouping of genotypes by multivariate methods allow us to choose elite lines from different clusters. The superior wheat genotypes identified were: 382 (with high SW, TGW, PC, and GC), 422 (with high TGW, PC, and GC), 630 (with high GY, PC, GC, and low PH), 696 (with high SW, PC, and GC), 721 (with high GY, GC, and low HT, PH) and 754 (with high GY, TGW, PC, GC, and low PH). All these genotypes were highly resistant to plant diseases examined.

## Conclusions

The present study represents the first comprehensive study of the variability of agronomic and qualitative traits in a large sample of *T. turanicum* collected from different eco-geographic regions around the world. In contrast to others tetraploid species, i.e., *T. dicoccoides* and *T. dicoccum,* the involvement of the *T. turanicum* in practical durum wheat breeding programs, by hybridization, still remains limited. Our results indicate that Khorasan wheat offer abundant genetic diversity for multiple agronomic adaptive traits and gives us the opportunity to exploit favorable alleles that were excluded from the domesticated genepool. Seven accessions were identified with a combination of desirable traits for high GY and other useful characteristics that can be further used in durum wheat breeding programs. Moreover, no studies were found in the literature that have considered the heritability, or phenotypic or genotypic correlations, for GY and its related traits, and the quality traits of lines derived from *T. durum* × *T. turanicum* crossing. The current research shows that the backcross strategy used is appropriate for transferring of novel alleles from wild relative species into durum wheat elite cultivars without a significant reduction in their valuable agronomic qualities. The promising introgressed lines have been selected for use in durum wheat breeding programs, based on their diverse and complementary agronomic traits that are adapted to the Mediterranean environment.

## Author Contributions

AI conceived, designed the experiment, analyzed the data, and wrote the paper. PC performed the experiments and recorded the data. All authors read and approved the final manuscript.

## Funding

This study was self-financed by the Council for Agricultural Research and Economics within the scope of a larger project supported by the Italian Ministry of Agricultural, Food and Forestry Policies.

## Conflict of Interest

The authors declare that the research was conducted in the absence of any commercial or financial relationships that could be construed as a potential conflict of interest.

## Supplementary Material

Figure S1 Heat map for Pearson*’*s correlation coefficients between the quantitative traits measured for the 77 Khorasan wheat accessions. Correlation coefficients were classified as weak (NS), moderate (P ≥0.05) and strong (P ≥0.01) (n=77).

## References

[B1] Allard R. W. (1960).Principles of Plant Breeding. New York: John Wiley and Sons.

[B2] Alsaleh A. Shehzad Baloch F. Nachit M. Ozkan H. (2016). Phenotypic and genotypic intra-diversity among Anatolian durum wheat ‘Kunduru’ landraces. Biochem. Syst. Ecol. 65, 9–16. 10.1016/j.bse.2016.01.008

[B3] Amiri R. Sasani S. Jalali-Honarmand S. Rasaei A. Seifolahpour B. Bahraminejad S. (2018). Genetic diversity of bread wheat genotypes in Iran for some nutritional value and baking quality traits. Physiol. Mol. Biol. Plants 24 (1), 147–157. 10.1007/s12298-017-0481-4 29398846 PMC5787113

[B4] Bekele E. (1984). Analysis of regional patterns of phenotypic diversity in the Ethiopian tetraploid and hexaploid wheats. Hereditas 100, 131–154. 10.1111/j.1601-5223.1984.tb00114.x

[B5] Bertan I. Carvalho F. I. F. Oliveira A. C. (2007). Parental selection strategies in plant breeding programs. J. Crop Sci. Biotechnol. 10, 211–222.

[B6] Blanco A. Simeone R. Gadaleta A. (2006). Detection of QTLs for grain protein content in durum wheat. Theor. Appl. Genet. 112 (7), 1195–1204. 10.1007/s00122-006-0221-6 16453131

[B7] Chatzav M. Peleg Z. Ozturk L. Yazici A. Fahima T. Cakmak I. (2010). Genetic diversity for grain nutrients in wild emmer wheat: potential for wheat improvement. Ann. Bot. 105 (7), 1211–1220. 10.1093/aob/mcq024 20202969 PMC2887062

[B8] Chen Y. Lübberstedt T. (2010). Molecular basis of trait correlations. Trends Plant Sci. 15, 454–461. 10.1016/j.tplants.2010.05.004 20542719

[B9] Crespel L. Le Bras C. Relion D. Morel P. (2014). Genotype × year interaction and broad-sense heritability of architectural characteristics in rose bush. Plant Breed. 133 (3), 412–418. 10.1111/pbr.12157

[B10] D’Egidio M. G. (2007). Overview on pasta in the world. Tecnica Molitoria Int. 58, 92–97.

[B11] De Vita P. Nicosia O. L. D. Nigro F. Platani C. Riefolo C. Di Fonzo N. (2007). Breeding progress in morpho-physiological, agronomical and qualitative traits of durum wheat cultivars released in Italy during the 20th century. Eur. J. Agron. 26 (1), 39–53. 10.1016/j.eja.2006.08.009

[B12] Ehdaie B. Waines J. G. (1989). Genetic variation, heritability, and path-analysis in landraces of bread wheat from South Western Iran. Euphytica 41, 183–190. 10.1007/BF00021584

[B13] Eticha F. Bekele E. Belay G. Börner A. (2005). Phenotypic diversity in tetraploid wheats collected from Bale and Wello regions of Ethiopia. Plant Genet. Resour. 3 (1), 35–43. 1079/PGR200457

[B14] Falconer D. S. Mackay T. F. C. (1996).Introduction to Quantitative Genetics. Harlow, Essex, UK: Longman (5th ed.).

[B15] FAO (2016).Climate change and food security: risks and responses. Rome: Food Agriculture Organization Available from: http://www.fao.org/3/a-i5188e.pdf.

[B16] González-Ribot G. Opazo M. Silva P. Acevedo E. (2017). Traits explaining durum wheat (*Triticum turgidum* L. spp. *durum*) yield in dry Chilean Mediterranean environments. Front. Plant Sci. 8, 1781. 10.3389/fpls.2017.01781 29104578 PMC5654942

[B17] Grausgruber H. Oberforster M. Ghambashidze G. Ruckenbauer P. (2005). Yield and agronomic traits of Khorasan wheat (*Triticum turanicum* Jakubz.). Field Crops Res. 91, 319–327. 10.1016/j.fcr.2004.08.001

[B18] Grenier C. Deu M. Kresovich S. Bramel-Cox P. J. Hamon P. (2000). Assessment of genetic diversity in three subsets constituted from the ICRISAT sorghum collection using random *vs* non-random sampling procedures. B. Using molecular markers. Theor. Appl. Genet. 101, 197–202. 10.1007/s001220051

[B19] Iannucci A. Codianni P. (2016). Effect of conventional and organic farming systems on bio-agronomic and quality traits of durum wheat under Mediterranean conditions. Aust. J. Crop Sci. 10 (8), 1083–1091. 10.21475/ajcs.2016.10.08.p7179

[B20] Jaradat A. A. Shahid M. A. (2006). Patterns of phenotypic variation in a germplasm collection of *Carthamus tinctorius* L. from the Middle East. Genet. Resour. Crop Evol. 53, 225–244. 10.1007/s10722-004-6150-9

[B21] Johnson H. W. Robinson H. F. Comstock R. E. (1955). Estimates of genetic and environmental variability in soybeans. Agron. J. 47, 314–318. 10.2134/agronj1955.00021962004700070009x

[B22] Jolliffe I. (2002). Principal Component Analysis. . New York: Springer.

[B23] Kamara A. Y. Kling J. G. Menkir A. Ibikunle O. (2003). Agronomic performance of maize (*Zea mays* L.) breeding lines derived from a low nitrogen maize population. J. Agric. Sci. 141 (2), 221–230. 10.1017/s0021859603003514

[B24] Khan M. A. Witzke-Ehbrecht S. Maass B. L. Becker H. C. (2009). Relationships among different geographical groups, agromorphology, fatty acid composition and RAPD marker diversity in safflower (*Carthamus tinctorius*). Genet. Resour. Crop Evol. 56, 19–30. 10.1007/s10722-008-9338-6

[B25] Khlestkina E. K. Röder M. S. Grausgruber H. Börner A. (2006). A DNA fingerprinting-based taxonomic allocation of Kamut wheat. Plant Genet. Resour. 4 (03), 172–180. 10.1079/pgr2006120

[B26] Laddomada B. Durante M. Mangini G. D’Amico L. Lenucci M. S. Simeone R. (2017). Genetic variation for phenolic acids concentration and composition in a tetraploid wheat (*Triticum turgidum* L.) collection. Genet. Resour. Crop Evol. 64, 587–597. 10.1007/s10722-016-0386-z

[B27] Laidò G. Mangini G. Taranto F. Gadaleta A. Blanco A. Cattivelli L. (2013). Genetic diversity and population structure of tetraploid wheats (*Triticum turgidum* L.) estimated by SSR, DArT and pedigree data. PloS ONE 8 (6), e67280. 10.1371/journal.pone.0067280 23826256 PMC3694930

[B28] Lopes M. S. El-Basyoni I. Baenziger P. S. Singh S. Royo C. Ozbek K. (2015). Exploiting genetic diversity from landraces in wheat breeding for adaptation to climate change. J. Exp. Bot. 66, 3477–3486. 10.1093/jxb/erv122 25821073

[B29] Mackay M. Street K. Hickey L. (2016). “Toward more effective discovery and deployment of novel plant genetic variation: reflection and future directions.” Applied mathematics and omics to assess crop genetic resources for climate change adaptive traits. Eds. Bari A. Damania A. B. Mackay M. Dayanandan S. (Boca Raton, FL, USA: Taylor & Francis Group), 139–150. 10.1201/b19518-16

[B30] Mansouri A. Oudjehih B. Benbelkacem A. Fellahi Z. E. A. Bouzerzour H. (2018). Variation and relationships among agronomic traits in durum wheat [*Triticum turgidum* (L.) Thell. ssp. *turgidum* conv. *durum* (Desf.) MacKey] under south Mediterranean growth conditions: stepwise and path analyses. Int. J. Agron. 2018, 8191749. 10.1155/2018/8191749

[B31] Mengistu D. K. Kidane Y. G. Fadda C. Pè M. E. (2016a). Genetic diversity in Ethiopian durum wheat (*Triticum turgidum* var *durum*) inferred from phenotypic variations. Plant Genet. Resour. 16 (1), 39–49. 10.1017/s1479262116000393

[B32] Mengistu D. K. Kidane Y. G. Catellani M. Frascaroli E. Fadda C. Pé M. E. (2016b). High-density molecular characterization and association mapping in Ethiopian durum wheat landraces reveals high diversity and potential for wheat breeding. Plant Biotechnol. J. 14, 1800–1812. 10.1111/pbi.12538 26853077 PMC5067613

[B33] Mohammadi R. Etminan A. Shoshtari L. (2018). Agro-physiological characterization of durum wheat genotypes under drought conditions. Exp. Agric. 2018, 1–16. 10.1017/S0014479718000133

[B34] Mwadzingeni L. Shimelis H. Tesfay S. Tsilo T. J. (2016). Screening of bread wheat genotypes for drought tolerance using phenotypic and proline analyses. Front. Plant Sci. 7, 1276. 10.3389/fpls.2016.01276 27610116 PMC4997044

[B35] Piergiovanni A. R. (2009). Estimating gliadin and albumin variation at intra- and interaccession level in USDA oriental wheat (*Triticum turgidum* L. subsp. *turanicum* (Jakubz.) (A. Löve & D. Löve) collection using capillary zone electrophoresis. Cereal Chem. 86, 37–43. 10.1094/CCHEM-86-1-0037

[B36] Rodríguez-Quijano M. Lucas R. Ruiz M. Giraldo P. Espí A. Carrillo J. M. (2010). Allelic variation and geographical patterns of prolamins in the USDA-ARS khorasan wheat germplasm collection. Crop Sci. 50, 2383–2391. 10.2135/cropsci2010.02.0089

[B37] Ross A. S. Bettge A. D. (2009). Passing the Test on Wheat End-Use Quality, in Wheat: Science and trade. Ed. Carver B. F. (Ames, IA, USA: Wiley-Blackwell Publishing), 455–493.

[B38] Royo C. Nazco R. Villegas D. (2014). The climate of the zone of origin of Mediterranean durum wheat (*Triticum durum* Desf.) landraces affects their agronomic performance. Genet. Resour. Crop Evol. 61, 1345–1358. 10.1007/s10722-014-0116-3

[B39] Ruiz M. Giraldo P. Royo C. Villegas D. Aranzana M. J. Carrillo J. M. (2012). Diversity and genetic structure of a collection of Spanish durum wheat landraces. Crop Sci. 52, 2262–2275. 10.2135/cropsci2012.02.0081

[B40] Salihu S. Grausgruber H. Ruckenbauer P. (2006). Agronomic and quality performance of international winter wheat genotypes grown in Kosovo. Cereal Res. Commun. 34 (2), 957–964. 10.1556/CRC.34.2006.2-3.225

[B41] Shakhatreh Y. Haddad N. Alrababah M. Grando S. Ceccarelli S. (2010). Phenotypic diversity in wild barley (*Hordeum vulgare* L. ssp. *spontaneum* (C. Koch) Thell.) accessions collected in Jordan. Genet. Resour. Crop Evol. 57, 131–146. 10.1007/s10722-009-9457-8

[B42] Sharma J. R. (1998).Statistical and Biometrical Techniques in Plant Breeding. New Delhi: New Age International Publication, 432.

[B43] Shewry P. R. Hey S. J. (2015). The contribution of wheat to human diet and health. Food Energy Secur. 4 (3), 178–202. 10.1002/fes3.64 27610232 PMC4998136

[B44] Sissons M. J. Hare R. A. (2002). Tetraploid wheat - a resource for genetic improvement of durum wheat quality. Cereal Chem. 79, 78–84. 10.1094/CCHEM.2002.79.1.78

[B45] Stagnari F. Codianni P. Pisante M. (2008). Agronomic and kernel quality of ancient wheats grown in central and southern Italy. Cereal Res. Commun. 36, 313–326. 10.1556/CRC.36.2008.2.11

[B46] Triboï E. Martre P. Girousse C. Ravel C. Triboï-Blondel A. M. (2006). Unravelling environmental and genetic relationships between grain yield and nitrogen concentration for wheat. Eur. J. Agron. 25, 108–118. 10.1016/j.eja.2006.04.004

[B47] Vavilov N. I. (1951). The origin, variation, immunity and breeding of cultivated plants (translated from the Russian by Chester K. S.), in Chronica Botanica 13 (Waltham, Mass: Chronica Botanica Co.).

[B48] Zadoks J. C. Chang T. T. Konzak C. F. (1974). A decimal code for the growth stages of cereals. Weed Res. 14, 415–421. 10.1111/j.1365-3180.1974.tb01084.x

[B49] Zanetti S. Winzeler M. Feuillet C. Keller B. Messmer M. (2001). Genetic analysis of breadmaking quality in wheat and spelt. Plant Breed. 120, 13–19. 10.1046/j.1439-0523.2001.00552.x

