# Supplementary figures and images for: Phenotypic Parent Selection Within a Khorasan Wheat Collection and Genetic Variation in Advanced Breeding Lines Derived by Hybridization With Durum Wheat
Source: Front Plant Sci. 2019 Nov 20;10:1460. doi: 10.3389/fpls.2019.01460 (PMC6903774; doi:10.3389/fpls.2019.01460)

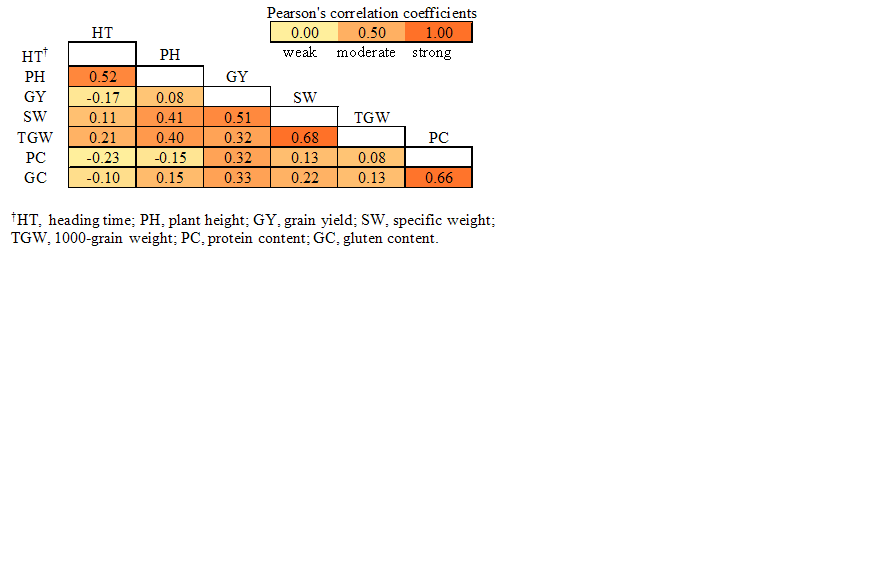

Supplement: Figure S1 — Heat map for Pearson’s correlation coefficients between the quantitative traits measured for the 77 Khorasan wheat accessions. Correlation coefficients were classified as weak (NS), moderate (P ≥0.05) and strong (P ≥0.01) (n=77). [file Image_1.tif]
