# Supplementary material for: Phenotypic Parent Selection Within a Khorasan Wheat Collection and Genetic Variation in Advanced Breeding Lines Derived by Hybridization With Durum Wheat
Source: Front Plant Sci. 2019 Nov 20;10:1460. doi: 10.3389/fpls.2019.01460 (PMC6903774; doi:10.3389/fpls.2019.01460)
Supplement: Supplementary file 2 [file DataSheet_1.docx]

**Table S1**. Breeding strategy (i.e. interspecific hybridization and backcrossing) used to obtain the 790 recombinant inbred lines (RILs) evaluated at Foggia (Italy) in 2013-2015.

| **Cross combination** | **RILs per cross** | **Superior line^†^** |
| --- | --- | --- |
| *T. durum* cv. Iride × *T. turgidum* subsp. *turanicum* PI 330552 | 74 |  |
| *T. durum* cv. PR22D89 × *T. turgidum* subsp. *turanicum* PI 330552 | 54 |  |
| *T. durum* cv. Iride × *T. turgidum* subsp. *turanicum* PI 525355 | 102 |  |
| *T. durum* cv. PR22D89 × *T. turgidum* subsp. *turanicum* PI 525355 | 52 | 382 |
| *T. durum* cv. Saragolla × *T. turgidum* subsp. *turanicum* PI 67343 | 18 |  |
| *T. durum* cv. Saragolla × *T. turgidum* subsp. *turanicum* PI 10391 | 16 |  |
| *T. durum* cv. Saragolla × *T. turgidum* subsp. *turanicum* PI 115815 | 23 |  |
| (*T. durum* cv. Iride × *T. turgidum* subsp. *turanicum* PI 330552) F_1_ × *T. durum* cv Iride | 11 |  |
| (*T. durum* cv. PR22D89 × *T. turgidum* subsp. *turanicum* PI 330552) F_1_ × *T. durum* cv PR22D89 | 10 |  |
| (*T. durum* cv. Iride × *T. turgidum* subsp. *turanicum* PI 525355) F_1_ × *T. durum* cv. Iride | 22 |  |
| (*T. durum* cv. PR22D89 × *T. turgidum* subsp. *turanicum* PI 525355) F_1_ × *T.durum* cv. PR22D89 | 15 |  |
| (*T. durum* cv. Saragolla × *T. turgidum* subsp. *turanicum* PI 67343 ) F_1_ × *T. durum* cv. Saragolla | 10 |  |
| (*T. durum* cv. Saragolla × *T. turgidum* subsp. *turanicum* PI 68287) F_1_ × *T. durum* cv. Saragolla | 11 | 422 |
| (*T. durum* cv. Saragolla × *T. turgidum* subsp. *turanicum* PI 10391) F_1_ × *T. durum* cv. Saragolla | 39 |  |
| (*T. durum* cv. Saragolla × *T. turgidum* subsp. *turanicum* PI 115815) F_1_ × *T. durum* cv. Saragolla | 18 |  |
| *(T. durum* cv. Iride × *T. turgidum* subsp. *turanicum* PI 330552) F_1_ × *T. durum* cv. Saragolla | 70 |  |
| (*T. durum* cv. PR22D89 × *T. turgidum* subsp. *turanicum* PI 330552) F_1_ × *T. durum* cv. Saragolla | 46 | 630 |
| (*T. durum* cv. Iride × *T. turgidum* subsp. *turanicum* PI 525355) F_1_ × *T. durum* cv. Saragolla | 78 | 696 and 721 |
| (*T. durum* cv. PR22D89 × *T. turgidum* subsp. *turanicum* PI 525355) F_1_ × *T. durum* cv. Saragolla | 59 | 754 |
| (*T. durum* cv. Iride × *T. turgidum* subsp. *turanicum* PI 330552) F_1_ × *T. turgidum* subsp. *turanicum* PI 330552 | 11 |  |
| (*T. durum* cv. PR22D89 × *T. turgidum* subsp. *turanicum* PI 330552) F_1_ × *T. turgidum* subsp. *turanicum* PI 330552 | 7 |  |
| (*T. durum* cv. Iride × *T. turgidum* subsp. *turanicum* PI 525355) F_1_ × *T. turgidum* subsp. *turanicum* PI 525355 | 17 |  |
| (*T. durum* cv. Iride × *T. turgidum* subsp. *turanicum* CLTR 14599) F_1_ × *T. turgidum* subsp. *turanicum* PI 525356 | 6 |  |
| (*T. durum* cv. PR22D89 × *T. turgidum* subsp. *turanicum* PI 525355) F_1_ × *T. turgidum* subsp. *turanicum* PI 525355 | 21 |  |

^†^Superior line for multiple traits as identified by cluster analysis (see Table 8).

**Table S2**. E*igenvalues, eigenvectors* and percentage of variation explained by the first three principal components assessed for 7 agronomic traits in 77 khorasan accessions grown at Foggia (Italy) over the three growing seasons (2005-2007).

|  |  | **Principal component axis** | | | | |  |
| --- | --- | --- | --- | --- | --- | --- | --- |
|  | **1** | | | **2** | **3** | | |
| *Eigenvalues* | *2.50* | | | *1.92* | *1.03* | | |
| PC variation (%) | 35.8 | | | 27.5 | 14.7 | | |
| Cumulative variation (%) | 35.8 | | | 63.3 | 78.0 | | |
| **Character**^†^ |  | | ***Eigenvectors*** | | |  | |
| HT (days) | 0.1139 | | | **0.5242** | 0.4572 | | |
| PH (cm) | 0.3392 | | | **0.4398** | 0.3289 | | |
| GY (t ha-1) | **0.4179** | | | -0.2381 | -0.3585 | | |
| SW (kg hl-1) | **0.5290** | | | 0.1152 | -0.3167 | | |
| TGW (g) | **0.4774** | | | 0.2086 | -0.2485 | | |
| PC (%) | 0.2619 | | | **-0.5206** | 0.3495 | | |
| GC (%) | 0.3480 | | | -0.3837 | **0.5197** | | |

^†^HT: days to heading from 1 April, PH: plant height, GY: grain yield, SW: specific

weight, TGW: thousand grain weight, PC: protein content, GC: gluten content.

The **bold** **values** are the most important traits for each factor axes.

**Table S3.** Mean values for the 7 agronomic traits for the six groups obtained by cluster analysis for the 77 khorasan accessions grown at Foggia (Italy) over the three growing seasons (2005-2007). Significant differences among the clusters at the 0.001 level was seen for all of the traits.

| **Trait** | **Cluster** | | | | | |
| --- | --- | --- | --- | --- | --- | --- |
|  | **I** | **II** | **III** | **IV** | **V** | **VI** |
|  | (n = 7) | (n = 22) | (n = 1) | (n = 5) | (n = 35) | (n = 7) |
| Heading time (days) ^†^*** | 32.10 | 34.20 | 26.00 | 32.73 | 32.18 | **35.33** |
| Plant height (cm)*** | 105.95 | **117.35** | 100.00 | 107.33 | 105.00 | 111.19 |
| Grain yield (t ha^-1^)*** | 3.09 | 3.11 | 3.44 | **3.49** | 2.69 | 1.71 |
| Specific weight (kg hl^-1^)*** | 80.60 | **81.08** | 75.77 | **81.12** | 75.72 | 72.22 |
| 1000-grain weight (g)*** | **60.95** | 58.95 | 42.03 | **60.95** | 49.86 | 48.91 |
| Protein content (%)*** | 13.21 | 13.27 | **15.30** | 14.20 | 13.35 | 12.88 |
| Gluten content (%)*** | 9.86 | 10.32 | **12.07** | 11.19 | 10.00 | 9.92 |

^†^ from April 1

Three asterisks represent 0.001 significant differences among clusters

Data in **bold text** indicate the highest value for each trait

**Table S4.** Mean and standard error (SE) for the 7 agronomic traits **o**f the selected genotypes of T*riticum turgidum* subsp. *turanicum* grown at Foggia (Italy) over the three growing seasons (2005-2007).

| **Selected genotype** | **Country of origin** | | **Trait**^†^ | | | | | | |
| --- | --- | --- | --- | --- | --- | --- | --- | --- | --- |
|  |  | | **HT** | **PH** | **GY** | **SW** | **TGW** | **PC** | **GC** |
| PI67343 | Australia | | 31.33 | 108.33 | **3.67** | **83.97** | **61.73** | 13.43 | 10.00 |
| CItr14599 | Ethiopia | | *26.00* | *100.00* | **3.44** | *75.77* | *42.03* | **15.30** | **12.07** |
| PI10391 | Egypt | | **34.00** | *93.33* | **3.75** | 76.57 | 52.43 | 13.97 | 9.77 |
| PI525355 | Morocco | | 33.33 | 105.00 | **4.20** | **83.40** | 58.70 | **14.03** | **11.03** |
| PI68287 | Azerbaijan | | **34.00** | 110.00 | 3.21 | **82.10** | **62.07** | **15.60** | **11.83** |
| PI115815 | Turkey | | 32.67 | 103.33 | **3.59** | **81.00** | **69.80** | 13.63 | 10.07 |
| PI330552 | United Kingdon | | 31.67 | 106.67 | **4.31** | 80.73 | 60.67 | **14.20** | 10.60 |
| **Mean ± SE** |  | | 31.9 ± 1.1 | 103.8 ± 2.1 | 3.7 ± 0.1 | 80.5 ± 1.2 | 58.2 ± 3.3 | 14.3 ± 0.3 | 10.8 ± 0.3 |
|  |  | |  |  |  |  |  |  |  |
| **Mean of the 77 Khorasan accessions** | |  | 33.00 | 109.26 | 2.82 | 77.73 | 54.00 | 13.35 | 10.17 |
| **SD^‡^ of the 77 Khorasan accessions** | |  | 1.87 | 6.50 | 0.58 | 3.64 | 7.00 | 0.56 | 0.53 |

^†^HT, days to heading from April 1; PH, plant height; GY, grain yield; SW, specific weight; TGW, 1000-grain weight; PC, protein content; GC, gluten content.

^‡^SD, standard deviation.

Data in **bold text** and in *italic text* indicate the highest and the lowest values for each trait, respectively.

**Table S5**. E*igenvalues, eigenvectors* and percentage of variation explained by the first

four principal components assessed for 11 traits in 800 genotypes, evaluated at Foggia (Italy) in 2013-2015.

|  |  | **Principal component axis** | | |  |  |
| --- | --- | --- | --- | --- | --- | --- |
|  | **1** | | **2** | **3** | | **4** |
| *Eigenvalues* | *2.42* | | *2.15* | *1.72* | | *1.40* |
| PC variation (%) | 22.0 | | 19.6 | 15.7 | | 12.8 |
| Cumulative variation (%) | 22.0 | | 41.5 | 57.2 | | 70.0 |
| **Character**^†^ |  | | ***Eigenvectors*** |  | |  |
| HT (days) | 0.2197 | | 0.2454 | 0.1989 | | 0.0504 |
| PH (cm) | **0.4053** | | -0.1414 | 0.3652 | | 0.0550 |
| GY (t ha^-1^) | -0.1331 | | **-0.4517** | -0.2966 | | 0.3124 |
| SW (kg hl^-1^) | 0.3806 | | -0.1650 | **0.4069** | | 0.1042 |
| TGW (g) | 0.2502 | | -0.3345 | 0.2333 | | **0.4054** |
| PC (%) | **0.4903** | | 0.0237 | -0.3625 | | -0.3062 |
| GC (%) | **0.4967** | | 0.0267 | -0.3636 | | -0.2825 |
| PM score | 0.1858 | | **0.4067** | -0.1930 | | **0.4257** |
| STB score | 0.0857 | | 0.1784 | -0.3668 | | **0.5787** |
| YR score | 0.0957 | | **0.4354** | 0.1761 | | 0.1543 |
| BR score | -0.1555 | | **0.4343** | 0.2295 | | -0.0909 |

^†^ HT: days to heading from 1 April, PH: plant height, GY: grain yield, SW: specific weight, TGW: thousand grain weight, PC: protein content, GC: gluten content, PM: powdery mildew resistance, STB: *Septoria tritici* blotch resistance, YR: yellow rust resistance, BR: brown rust resistance.

The **bold values** are the most important traits for each factor axes.
